# A unified approach to sequential and non-sequential structure alignment of proteins, RNAs, and DNAs

**DOI:** 10.1016/j.isci.2022.105218

**Published:** 2022-09-28

**Authors:** Chengxin Zhang, Anna Marie Pyle

**Affiliations:** 1 Department of Molecular, Cellular and Developmental Biology, Yale University, New Haven, CT 06511, USA; 2 Howard Hughes Medical Institute, Chevy Chase, MD 20815, USA; 3 Department of Computational Medicine and Bioinformatics, University of Michigan, Ann Arbor, MI 48109, USA; 4 Department of Chemistry, Yale University, New Haven, CT 06511, USA

**Keywords:** Biological sciences, Molecular biology, Molecular structure

## Abstract

Many distantly related structure pairs exhibit structural similarities that can only be fully captured by a non-sequential alignment program. We present US-align2, a unified protocol for both sequential and non-sequential alignment of proteins and nucleic acids. On manually curated reference alignments for protein structural pairs with non-sequential relations, US-align2 achieves ≥13% higher agreement with reference alignments than existing sequential and non-sequential alignment methods. Non-sequential alignments also enabled US-align2 to have higher sensitivities in detecting RNA pairs from the same family with sequence identities <40%, obtaining ≥9% higher area under the receiver operating characteristic curve than third-party programs. The unique ability of US-align2 to parse both proteins and nucleic acids allows the method to detect protein-RNA and protein-DNA mimicries. Additionally, US-align2 performs full and semi-non-sequential alignments with at least 48% and 14% faster speed than existing programs for the same tasks, making it particularly useful for large-scale structural similarity detection.

## Highlights

• US-align2 performs sequential and non-sequential (NS) tertiary structure alignment
• It achieves 13% higher NS protein alignment accuracy than other methods
• US-align2 allows more sensitive detection of RNAs from the same Rfam family
• US-align2 validates existing protein-RNA and protein-DNA mimicries

## Graphical abstract

**Figure:**
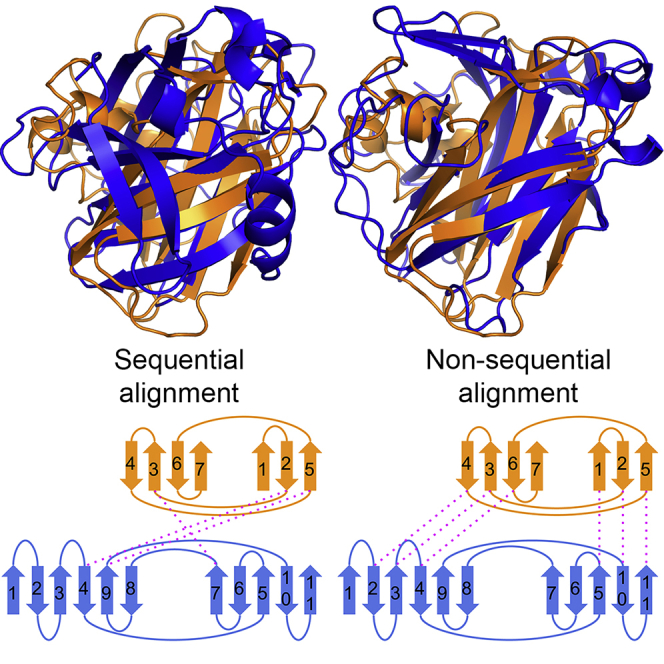

## Introduction

Structural similarities often imply functional similarities and evolutionary relatedness, and therefore many programs have been developed to perform tertiary structure alignments between pairs of macromolecules. Some of the commonly used structure alignment methods include DALI (Holm and Sander, 1993), CE (Shindyalov and Bourne, 1998), SPalign (Yang et al., 2012), and TM-align (Zhang and Skolnick, 2005) for proteins, as well as RMalign (Zheng et al., 2019), ARTS (Dror et al., 2006), and RNA-align (Gong et al., 2019) for RNAs. These programs align a pair of structures in sequential order. Mathematically, in a sequential (SQ) alignment, for any residue pair *i* and *j* from Structure A that are aligned with residues *i*’ and *j*’ in Structure B, respectively, where *i* < *j*, *i*' is always < *j*’.

While this sequentiality condition allows for efficient implementation by dynamic programming, it also makes the resulting alignment less relevant for pairs of molecules with a 3D architectural similarity that can only be described by a non-sequential relationship. In fact, it was previously estimated that rearrangement of fragments that can only be identified through non-sequential (NS) alignment is present in at least 17.4% of all structurally similar protein pairs (Abyzov and Ilyin, 2007). The most frequently reported NS alignments are for circular permutation, where the N-terminal portion of one protein is aligned to the C-terminal portion of another protein. More sophisticated NS cases also exist where structure fragments are swapped without circular permutation (Abyzov and Ilyin, 2007). Moreover, in certain local structure contexts, such as a comparison of binding interfaces (Brylinski, 2014) or in the case of molecular mimicry between proteins and nucleic acids (Cui et al., 2015), NS alignment is preferred over SQ alignment, as we are more interested in the similarity of overall shape regardless of sequentially.

There are two main types of algorithms for NS alignment. The first is a semi-non-sequential (sNS) alignment, which preserves the sequential order within aligned fragment pairs, usually being secondary structure elements, while regions connecting these fragments pairs are aligned non-sequentially. Typical methods in this category include GANGSTA+ (Guerler and Knapp, 2008), MASS (Dror et al., 2003), and FlexSnap (Salem et al., 2010). The second type is a fully non-sequential (fNS) alignment, also known as a sequence-order-independent alignment. In fNS alignment, the atomic structure of a protein backbone is treated as a cloud of points that lack any sequence order information. The goal of fNS is to assign points from one cloud to the other cloud in order to maximize the structural overlap between the two clouds of points. Representative fNS algorithms for full-length proteins include SPalignNS (Brown et al., 2016), CLICK (Nguyen et al., 2011), and SAMO (Chen et al., 2006), while eMatchSite (Brylinski, 2014) and PROSTA-inter (Cui et al., 2015) are for local alignment of binding interfaces.

Despite previous advances in NS alignment of protein structures, many challenges remain. First, there is no NS algorithm for full-length alignment of RNAs or DNAs. Second, there is no available NS algorithm for the alignment of different biomolecular types. For example, protein and nucleic acid molecules cannot be aligned for quantitative molecular mimicry detection. Third, due to much larger search space, an NS alignment program is typically at least twice if not several times slower than a SQ alignment program that uses a similar scoring function. This makes large-scale NS alignment computationally prohibitory.

To address these challenges, we present US-align2, which performs SQ, sNS, and fNS structure alignment for both proteins and nucleic acids using a unified scoring function, i.e., the TM-score (Gong et al., 2019; Zhang and Skolnick, 2004), which is independent of molecule length. US-align2 is an extension of US-align (Zhang et al., 2022), which we previously developed for SQ alignment of proteins, nucleic acids, and macromolecular complexes. The US-align2 algorithm not only offers a faster and more accurate NS alignment with greater structure overlap than previous methods but it is also the first method for NS alignment of full-length RNAs. Additionally, US-align2 implements all functionalities of the original US-align program.

## Results

### Non-sequential alignment of hard-to-align protein structure pairs

US-align2 and eight existing programs for NS and SQ alignments were first tested on the RIPC dataset (Mayr et al., 2007) of pairwise protein structure alignment. Different from many existing datasets for reference alignments of protein structures, such as HOMSTRAD (Mizuguchi et al., 1998), FSSP (Holm et al., 1992), and SABmark (Van Walle et al., 2005), which were generated by automated protein SQ structure alignment programs, the RIPC reference alignments are manually curated. Additionally, the protein pairs from RIPC reference alignments are hard to align due to repetitions, large insertions/deletions, circular permutations, and/or conformational changes. The expert curations and specific focus on NS relation among structure pairs make the dataset ideal for testing NS methods. Similar to a previous study (Brown et al., 2016), three reference alignments were excluded as they align pairs of proteins with identical sequences. The remaining 20 reference alignments were for protein pairs with sequence identity <30%.

As per the previous study (Brown et al., 2016), performance was measured from both reference-dependent and reference-independent metrics. There are two reference-dependent metrics. The first is equivalent reference residue (EQR), which is the total number of aligned residue pairs shared by the manually curated reference alignment and the automated alignment from a structure alignment program. The second reference-dependent metric is percentage of agreement, which equals to EQR divided by the length of the reference alignment. Reference-independent metrics included the number of aligned residues (*L*_*ali*_), root mean square deviation (RMSD) of aligned residues, running time, and structure overlap (*SO*), which is defined as the percentage of residues aligned within 3.5Å to corresponding residues in the other structure:

(Equation 1) $$SO=\frac{100\%}{\text{min}\left\{L_{A},L_{B}\right\}}\sum_{i=1}^{L_{ali}}I\left[d_{i}<3.5\right]$$

**Figure 1:**
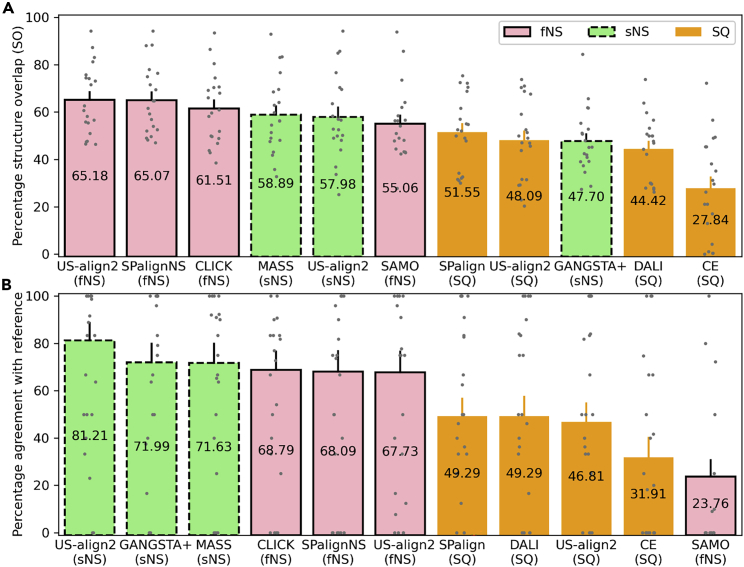
Performance of protein structure alignment by 11 different methods The bar height represents (A) the average structure overlap or (B) the total agreement with reference alignment. The error bar shows the standard error of mean (SEM). Each individual gray dot represents one pairwise alignment (n = 20). The performance for TM-align is not list separately as it generates identical alignments as US-align2 SQ.

Here, *L*_*A*_ and *L*_*B*_ are the sequence length of the two proteins, *d*_*i*_ is the distance of the i-th aligned residue pair, and *I*[ ] is the Iverson bracket, which equals to 1 if *d*_*i*_<3.5 and 0 otherwise. Since *SO* considers both the alignment coverage and deviation at the aligned region, it is a more useful reference-independent metric than *L*_*ali*_ and RMSD, which often conflict with each other. For example, the alignment program with the lowest RMSD (1.76 Å) was MASS, which also had the smallest *L*_*ali*_ (116) (Table 1). At the other end of the spectrum was CE, which had both the greatest *L*_*ali*_ (205) among all programs and the highest RMSD (18.35 Å). Neither program had the best *SO*. The two programs with the worst *SO* values (CE and DALI) both performed SQ alignment, while the three programs with the highest *SO* values were all fNS methods (US-align2 at fNS mode, SPalignNS, and CLICK) (Figure 1A). This is understandable, as NS alignment, especially fNS alignment, allows optimization of the alignment in a search space that is much larger than SQ alignment. Overall, US-align2 under the fNS mode has the highest SO (65.18%), which was 12.4% and 35.5% higher than US-align2 at sNS and SQ mode, respectively.

**Table 1:** Summary of protein structure alignment for the RIPC dataset

Method	*L*_ali_^a^	RMSD (Å)	SO (%)	EQR	Agreement (%)	Time (s)
US-align2 (SQ)	135	3.47	48.09	132	46.8	**0.164**
US-align2 (sNS)	168	3.57	57.98	**229**	**81.2**	0.369
US-align2 (fNS)	179	3.30	**65.18**	191	67.7	0.765
SPalign (SQ)	123	2.67	51.55	139	49.3	0.497
SPalignNS (fNS)	130	1.91	65.07	192	68.1	1.459
CLICK (fNS)	123	1.97	61.51	194	68.8	2.597
MASS (sNS)	116	**1.76**	58.89	202	71.6	0.431
GANGSTA+ (sNS)	126	2.83	47.70	203	72.0	0.745
SAMO (fNS)	156	2.97	55.06	67	23.8	2.580
DALI (SQ)	153	9.25	44.42	139	49.3	1.596
CE (SQ)	**205**	18.35	27.84	90	31.9	0.207

a Evaluation metrics that are independent of reference alignment are shown as average values, including *L*_*ali*_, RMSD, SO and time. On the other hand, metrics that are dependent on reference alignment are shown as the total value, including EQR and agreement, as per a previous study (Brown et al., 2016). This is because most reference alignments in the RIPC dataset contain a very small number of residue pairs (≤10) manually confirmed by the original author based on sequence and function conservation. Therefore, the average values for reference-based evaluation can be heavily biased by a few aligned residue pairs from the short reference alignments. For each metric, the value from the best program is highlighted in bold.

Nonetheless, a high *SO* alignment is not necessary biologically relevant. For example, although the fNS program SAMO had a reasonable *SO* (55.06%), which was higher than any of the four SQ alignment programs in this benchmark, its alignment had the worst agreement (23.76%) with the reference alignments manually curated according to evolutionary and functional insights (Figure 1B). In fact, although fNS appears to generate higher *SO* alignments than sNS, all three programs that agree well with the reference alignment were operating under the sNS mode rather than the fNS mode. Among them, US-align2 sNS had the highest agreement with the reference (81.21%), which is ∼13% higher than the second (GANGSTA+) and third place (MASS) programs. These data suggest that US-align2 sNS alignments may have higher biological relevance than US-align2 fNS alignments, even though the latter had better apparent structural overlaps.

In terms of speed, NS alignment is generally slower than SQ alignment using a similar objective function. For example, SPalignNS, which extended the original SQ alignment method SPalign for NS alignment, was almost three times slower than SPalign (Table 1). This is also true for US-align2, whose SQ alignment mode was on average 4.7 and 2.3 times faster than its fNS and sNS modes, respectively. Nonetheless, thanks to the fast heuristic alignment-superimposition iterations implemented in US-align2 (See STAR Methods), it was faster than any other programs in the same alignment mode. US-align2 fNS, sNS, and SQ used 47.6%, 14.4%, and 20.8% less time than SPalignNS, MASS, and CE, respectively, which were the second fastest programs for fNS, sNS, and SQ alignments in our benchmark. This makes US-align2 suitable for large-scale structure analysis. Note that the running time of a program depends on the hardware. For all benchmarks in this study, all programs are run single-threaded on the Yale Grace supercomputer equipped with the Intel Xeon Gold 6240 CPUs.

### Detecting protein pairs from the same structure fold by non-sequential alignment

To further evaluate the ability of different structure alignment program to differentiate protein pairs from the same versus different folds, we collect a large dataset of 954 protein domains from the ASTRAL40 database version 2.08, which is a non-redundant subset of the Structural Classification of Proteins–extended (SCOPe) database with pairwise sequence identity <40%. To collect this dataset, we first only kept the structure with the best resolution in each protein superfamily in the ASTRAL40 set. We then removed proteins from all SCOPe folds with less than two superfamilies, resulting in 954 proteins, each from a different superfamily, that belong to 146 SCOPe folds.

**Figure 2:**
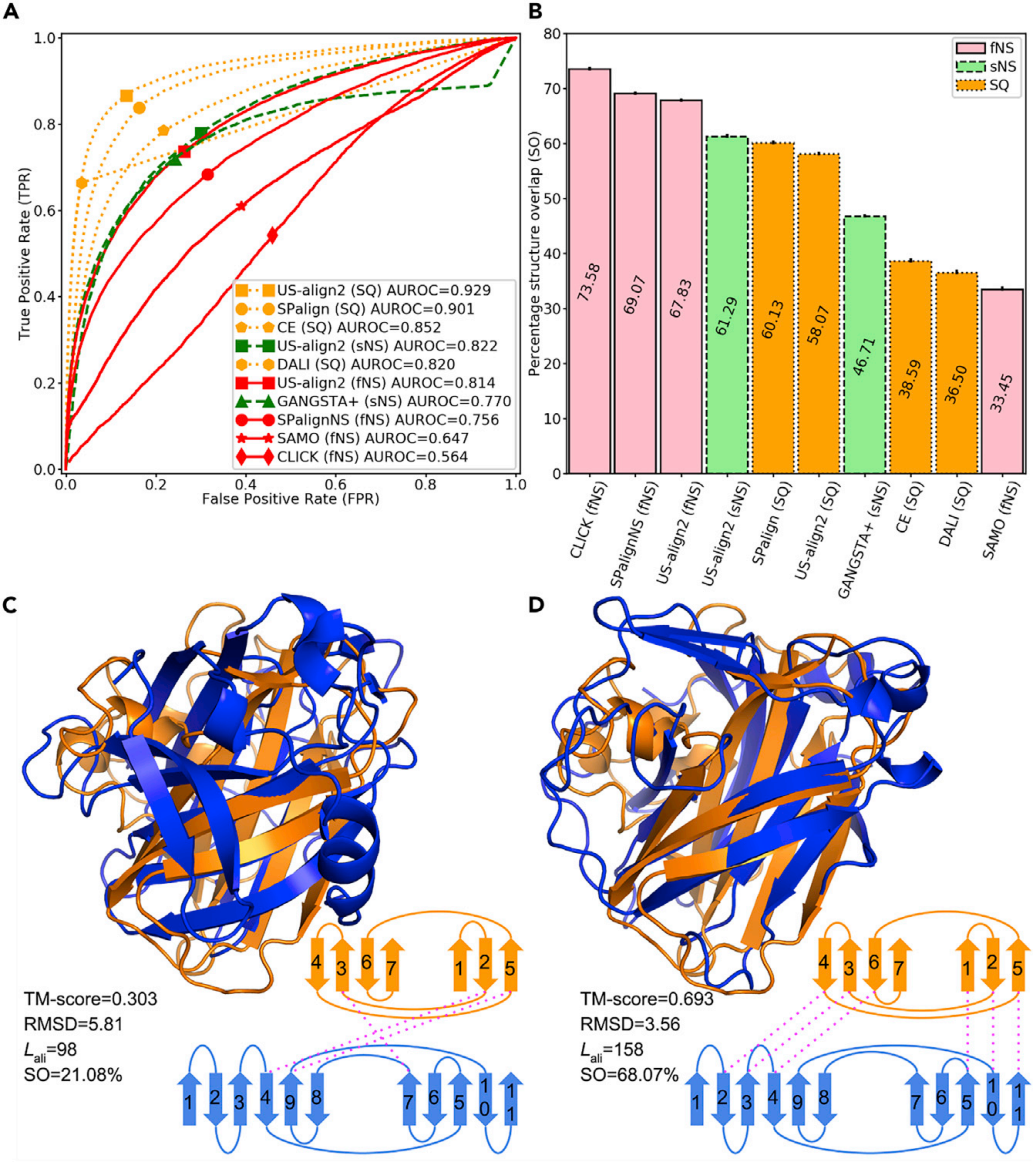
SCOPe fold detection by protein structure alignment (A) ROC of SCOPe family detection for all n = 454581 pairs of structures. Since US-align2 reports two TM-scores per RNA pairs, each score normalized by one of the two RNAs, the average of the two TM-scores was used to draw the curves for US-align2. (B) Mean structure overlap of all n = 7461 pairs of proteins from the same SCOPe fold. The error bar shows the standard error of mean (SEM). (C-D) US-align2 alignment between SCOPe: d4a02a_ (orange) and SCOPe: d4qi3a1 (blue) by (C) SQ alignment and (D) and sNS alignment. Schematic of the β-sheets for the two proteins are shown at lower right, where the β-strands are numbered from N- to C-terminal. Aligned β-strands are connected by pink dotted lines. US-align2 fNS alignment is not shown separately for this protein pair because it is the same as the sNS alignment. TM-scores are normalized by the shorter protein (d4a02a_).

All-against-all alignments were performed on this dataset by US-align2 and third-party protein alignment programs. All structure pairs were then sorted in descending order of alignment scores (e.g., TM-score for US-align2 and SPscore for SPalign), except for SAMO alignments, which were sorted in ascending order of the alignment score because a lower (i.e., more negative) SAMO score indicates a higher structural similarity. Each pair was labeled positive or negative depending on whether the pair shared the same SCOPe fold or not. Any self-hit (i.e., alignment of one protein to itself) was not considered. The performance of Rfam family detection at an alignment score cutoff *c* was quantified by the true positive rate (TPR) and false positive rate (FPR):

(Equation 2) $$\begin{array}{l} TPR\left(c\right)=\frac{TP\left(c\right)}{P}\\ FPR\left(c\right)=\frac{FP\left(c\right)}{N} \end{array}\;$$

where true positive *TP*(*c*) and false positive *FP*(*c*) were the number of positive and negative pairs with alignment score ≥*c* (or ≤*c* in the case of SAMO), respectively, while *P* and *N* were the total number of positive and negative RNA pairs. The receiver operating characteristic (ROC) curve could then be drawn for TPR versus FPR at all possible alignment score cutoffs (Figure 2A). The area under ROC curve (AUROC) summarized the ability of the alignment score to differentiate positive from negative protein pairs, where a perfect method would have AUROC = 1.

This benchmark shows that SQ alignment methods are usually more capable than NS methods to detect protein pairs from the same fold (Figure 2A). Among all methods, US-align2 SQ has the best AUROC (0.929) followed closely by SPalign (0.901), although NS methods usually produce alignments with better structure overlaps (Figure 2B). The most extreme case is the fNS method CLICK, which has the best average structure overlap of 73.58% but the worst AUROC (0.564), which is close to random results (AUROC = 0.5). These data are not surprising given that only a minority (17.4%) of pairs of topologically similar protein structures have NS relations (Abyzov and Ilyin, 2007), in contrast to RNA structure pairs where NS relations are more common as shown in a later section.

Despite having a lower precision, NS alignment can sometimes detect structural similarity that SQ alignment is not sensitive enough to identify. For example, both the cellobiose dehydrogenase and the chitin monooxygenase (SCOPe: d4qi3a1 and SCOPe: d4a02a_, respectively) share the same immunoglobulin-like beta-sandwich fold with complicated topology. US-align2 SQ is only able to align 3 out of the 7 β-strands of d4a02a_ with a poor TM-score of 0.303 (Figure 2C). On the other hand, US-align sNS alignment produces a completely different superimposition and a much higher TM-score (0.693), which is well above the cutoff for significant structure similarity (TM-score≥0.5). This is because the sNS alignment can discard the connectivities among the β-strands, enabling the alignment of 6 out of all 7 strands from d4a02a_ to be aligned (Figure 2D).

### Detecting related RNA pairs by non-sequential alignment

In this section, NS and SQ alignment modes of US-align2 were further benchmarked for homologous RNA detection on the Rfam dataset. To construct this dataset, sequences of all 7668 RNA PDB chains with at least one Rfam family match in Rfam database version 14.8 (Kalvari et al., 2021) were extracted from their PDB coordinates. Sequence redundancies among the RNAs were then removed by CD-HIT-EST (Huang et al., 2010), resulting in a final set of 508 representative RNAs mapped to 126 Rfam families, where any two RNAs shared <80% sequence identity. We then repeat the same ROC analysis for all-against-all alignments on this dataset by US-align2 under fNS, sNS, and SQ modes, as well as by two existing RNA alignment programs (RMalign and ARTS). Each pair was labeled positive or negative depending on whether the pair shared at least one identical Rfam family or not.

**Figure 3:**
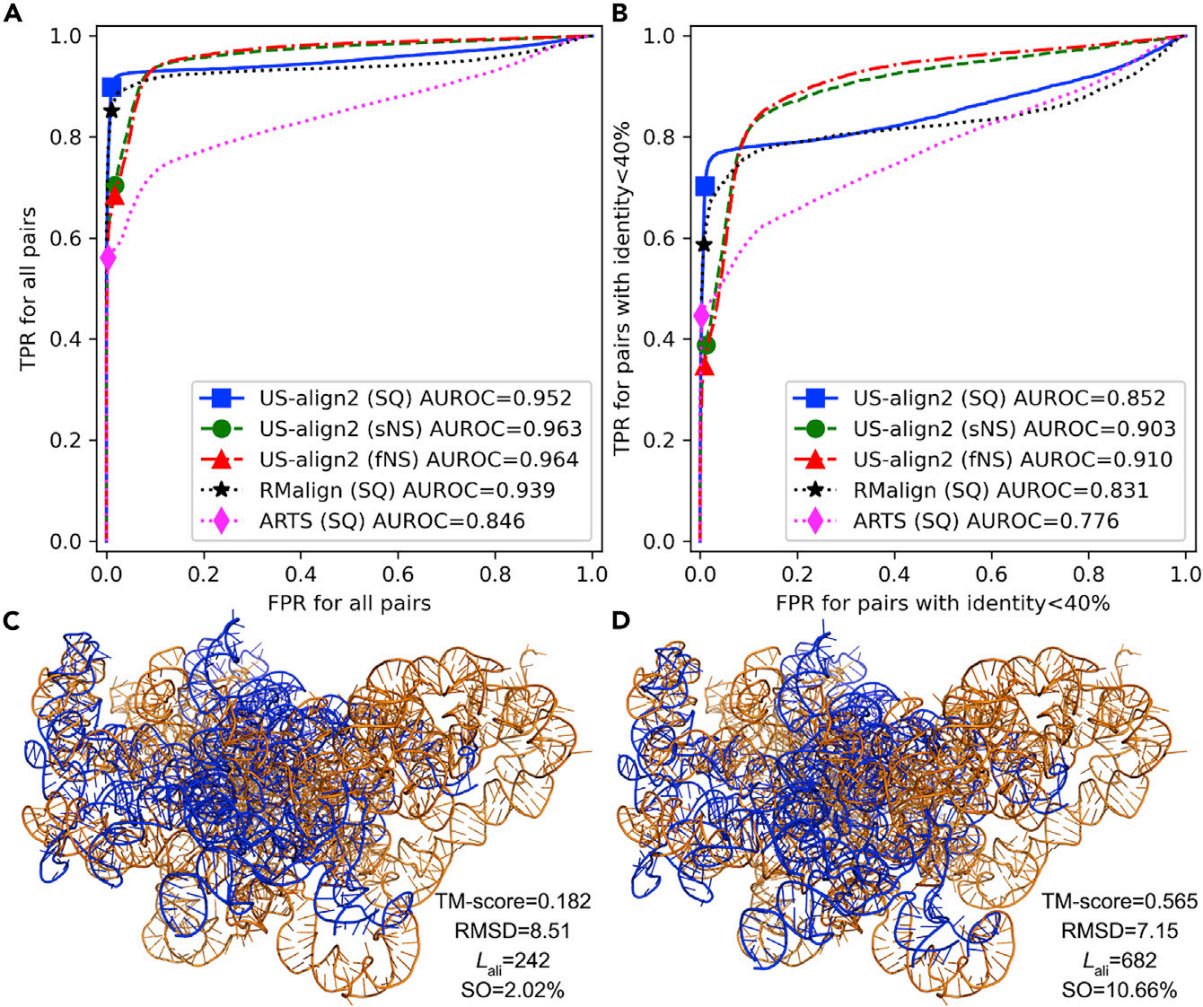
Rfam family detection by RNA structure alignment (A and B) ROC curves for Rfam family detection for (A) all n = 128778 RNA pairs and (B) the subset of n = 21813 RNA pairs with <40% sequence identity. The sequence identity equals to the number of identical residues in a US-align2 sequential alignment, divided by the length of the shorter of the two RNAs in the RNA pair. Since US-align2 reports two TM-scores per RNA pairs, each score normalized by one of the two RNAs, the average of the two TM-scores was used to draw the curves for US-align2. Similarly, the average of the two RMscores per RNA pairs was used to draw the curves for RMalign. (C-D) US-align2 alignment between 6em1 Chain 1 (orange) and 6az3 Chain 2 (blue) by (C) SQ alignment and (D) sNS alignment.

As shown in Figure 3A, US-align2 fNS and sNS both had ∼1.2% higher AUROC than US-align2 SQ, which in turn had 1.4% and 12.5% higher AUROC than third-party programs RMalign and ARTS, respectively. It may appear that the differences between the AUROCs of US-align2 NS alignments and those of US-align2 SQ and RMalign are small in absolute values. This is because the majority (68.5%) of RNA pairs from the same Rfam family are close homologs with ≥40% sequence identities. Since the list of top alignment hits is dominated by close homologs that are easy to align by all top performing programs, almost all programs, except for ARTS, have near perfect AUROC scores that are close to 1.

To evaluate the abilities of different programs to compare distant homologs that are difficult to align, the same benchmark is repeated for the subset of 21813 RNA pairs with <40% sequence identities (Figure 3B). More pronounced differences in performances are observed, where the AUROC of US-align2 fNS and sNS was 6.8% and 6.0% higher than US-align2 SQ, 9.5% and 8.7% higher than RMalign, and 17.3% and 16.4% higher than ARTS, respectively. These data demonstrate the utility of NS alignment for detecting related RNA pairs, especially when the sequence similarity is low.

An example of RNA pairs whose structural similarity is recognized only by NS alignment is the eukaryotic large subunit ribosomal RNA (Rfam: RF02543) from baker’s yeast and *Leishmania* (PDB: 6em1 Chain 1 and PDB: 6az3 Chain 2, respectively). Although both rRNAs belong to the same Rfam family and perform the same function, US-align2 SQ alignment only reports a very low TM-score (0.182, Figure 3C). RMalign also reports a very low similarity with RMscore 0.153, where RMscore≥0.5 is the cutoff for significant structural similarity (Zheng et al., 2019). The consistent failure of structural similarity detection by SQ alignment is due to the yeast rRNA being in an intermediate state during ribosome assembly (Kater et al., 2017) while the conformation of the *Leishmania* rRNA corresponds to the state in the final assembled ribosome (Shalev-Benami et al., 2017). Despite their conformational differences, US-align2 sNS can identify the structural similarity at TM-score 0.565 (Figure 3D) which is well above the cutoff for a pair of structurally related RNAs (TM-score≥0.45).

These data, however, do not suggest that NS alignment should always be preferred over SQ alignment for RNAs. Although both modes of NS alignments by US-align2 had higher TPRs than SQ programs at the high FPR region (FPR≥0.1), their TPRs were below US-align2 SQ in the low FPR region (FPR<0.1) (Figure 3). This implies that although NS alignment modes of US-align2 are less precise than US-align2 SQ for the very top hits (corresponding to the low FPR region), they are more sensitive than US-align2 SQ as more hits are considered (corresponding to the high FPR region).

Even though RMalign and ARTS both performed SQ alignment rather than the more computationally expensive NS alignment, they still required 0.74 and 0.32 second per alignment, respectively. This was at least 8.0 and 3.6 times slower than US-align2 under sNS and fNS modes, which takes 0.04 and 0.09 second per alignment, respectively. Although not as fast as US-align2 SQ at 0.02 second per alignment, US-align2 sNS and fNS were still among the fastest alignment programs for RNA tertiary structures.

### Case studies for molecular mimicries validated by non-sequential structure alignment

The ability of US-align2 to handle both proteins and nucleic acids makes it an ideal program for identifying protein-RNA molecular mimicry. Such mimicry between a protein and a nucleic acid molecule usually implies similarity in overall shape but not consistency in sequence order. Therefore, NS alignment would be more relevant than SQ alignment for detecting such cases.

**Figure 4:**
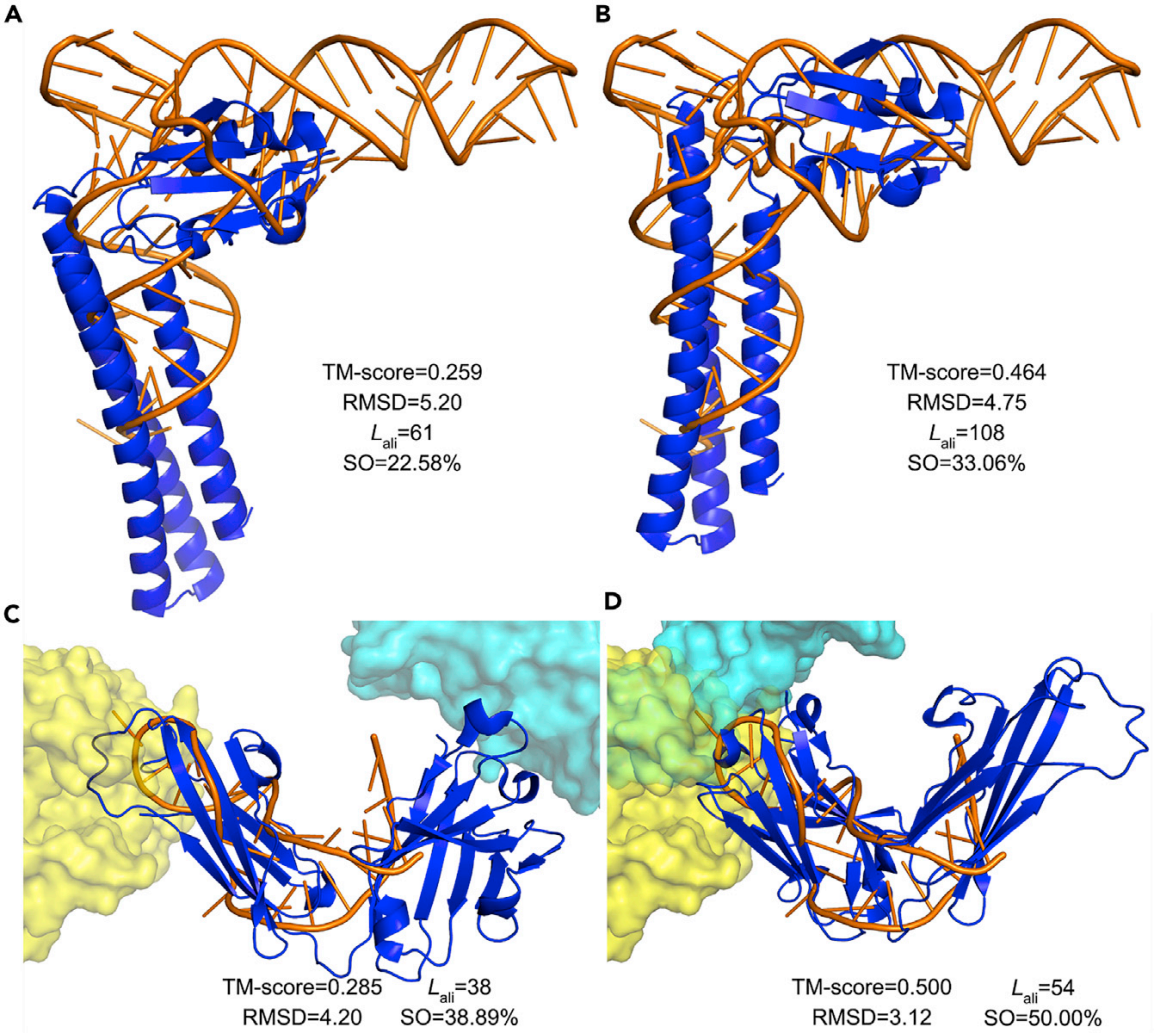
RNA-protein and DNA-protein mimicries validated by NS alignment (A and B) US-align2 alignment between the RRF protein (blue, PDB: 1eh1 Chain A) and tRNA^Phe^ (orange, PDB: 1evv Chain A) using (A) SQ and (B) fNS alignment. (C and D) US-align2 alignment between an antibody heavy chain (blue, PDB: 1eo8 Chain H) and a DNA aptamer (orange, PDB: 3zh2 Chain E) using (A) SQ alignment and (B) fNS alignment. The yellow and cyan surfaces are hemagglutinin (PDB: 1eo8 Chain A) and lactate dehydrogenase (PDB: 3zh2 Chain B), which are recognized by the antibody and the DNA aptamer, respectively. Since a nucleotide residue is much bigger than an amino acid residue in size, a nucleotide is represented by two atoms (P and C4’) while an amino acid is represented by one atom (Cα) when aligning the RNA to the protein, as implemented by the US-align2 option -atom “PC4’”. These atoms were chosen because the distance between a P and the adjacent C4’ atom coincides with the distance between two adjacent Cα atoms at approximately 3.8 Å. TM-scores in all panels were normalized by the length of the nucleic acid, as it is shorter than the protein.

For example, the ribosome recycling factor (RRF) protein and the tRNA is a pair of known molecular mimicry (Selmer et al., 1999). Both molecules are L-shaped and both bind to the same site within the ribosome. A tRNA transports an amino acid into an mRNA-bound ribosome to enable translation, while the RRF terminates translation by releasing the mRNA from the ribosome. Under SQ mode, US-align2 poorly captured the structure similarity, with an insignificant TM-score of 0.259, where the alignment only covers 49.2% of RNA nucleotides. The two molecules were poorly superimposed visually, with a large portion of RRF extruding outside the aligned region to the tRNA (Figure 4A). On the other hand, US-align2 NS aligned this pair of structures much better, as the superimposition placed RRF almost entirely within the envelop of the tRNA, where the alignment covers 87.1% of the nucleotides (Figure 4B). The TM-score is 0.464, which is above the TM-score cutoff of 0.45 to consider the RNA alignment to be significant (Gong et al., 2019).

Molecular mimicry also occurs for DNA aptamers, which are DNA oligonucleotides that fold into unusual tertiary structures to recognize protein epitopes in a similar fashion as antibody-epitope binding (Ren et al., 2021). Such protein-DNA mimicries cannot be easily detected by SQ alignment. For example, US-align2 SQ reports a low TM-score of 0.285 between the antibody for hemagglutinin and the DNA aptamer of lactate dehydrogenase. The regions for epitope recognition by the antibody and the aptamer are completely unaligned in SQ (Figure 4C). On the other hand, US-align2 NS reports a significant TM-score of 0.500 and produces superpositions that overlays the epitope recognition sites well (Figure 4D).

## Discussion and conclusion

In this work, we present the US-align2 algorithm for both SQ and NS alignment. The NS alignment modes enable accurate identification of non-sequential residue correspondence between protein structure pairs and the sensitive detection of remote RNA homologs. Such sensitivities are achieved at a speed several times faster than existing programs. The unique ability of US-align2 to align proteins with nucleic acids under a unified scoring function (TM-score) enables it to quantitatively capture a protein-RNA mimicry.

### Limitations of the study

At present, US-align2 does not consider conformational variations between a pair of related structures. Future development of US-align2 will focus on the extension to flexible alignment.

## STAR★Methods

### Key resources table

**Table:** 

REAGENT or RESOURCE	SOURCE	IDENTIFIER
**Deposited data**		

Benchmark dataset of US-align2	figshare	https://doi.org/10.6084/m9.figshare.20102945.v2

**Software and algorithms**		

SPalign	(Yang et al., 2012)	https://sparks-lab.org/downloads/
SPalignNS	(Brown et al., 2016)	https://sparks-lab.org/downloads/
CLICK	(Nguyen et al., 2011)	http://cospi.iiserpune.ac.in/click/
MASS	(Dror et al., 2003)	http://bioinfo3d.cs.tau.ac.il/MASS/
GANGSTA+	(Guerler and Knapp, 2008)	https://github.com/guerler/gplus
SAMO	(Chen et al., 2006)	http://doc.aporc.org/wiki/Samo
DALI	(Holm and Sander, 1993)	http://ekhidna2.biocenter.helsinki.fi/dali/
CE	(Shindyalov and Bourne, 1998)	http://ce.sdsc.edu/ce.html
RMalign	(Zheng et al., 2019)	http://rnabinding.com/RMalign/RMalign.html
ARTS	(Dror et al., 2006)	http://bioinfo3d.cs.tau.ac.il/ARTS/
US-align2	This paper	https://github.com/pylelab/USalign

### Resource availability

#### Lead contact

Further information and requests for resources should be directed to and will be fulfilled by the Lead Contact, Anna Marie Pyle (anna.pyle@yale.edu).

#### Materials availability

This study did not generate new unique reagents.

#### Data and code availability

• Structure files for datasets used in this study are available online at https://doi.org/10.6084/m9.figshare.20102945.v2.
• The source code of US-align2 version 20220606 used in this study is available at https://doi.org/10.6084/m9.figshare.20102945.v2. The latest version of US-align2 source code is available at https://github.com/pylelab/USalign.
• Any additional information required to reanalyze the data reported in this work paper is available from the lead contact upon request.

### Method details

#### TM-score

US-align2 quantifies the similarity between two structures by TM-score:

(Equation 3) $$TM=\frac{1}{L}\sum_{i=1}^{L_{ali}}\frac{1}{1+{\left(d_{i}/d_{0}\right)}^{2}}\;$$

Here, *d*_*i*_ is the distance between the Cα atoms for the *i*-th aligned amino acid residue (or C3’ atoms for nucleotide residues), *L*_*ali*_ is the number of aligned residues, and *L* is the length, i.e., the total number of residues in the structure. Since the two structures in an alignment may have two different lengths, each pairwise alignment can have two different TM-scores depending on which length is used for TM-score normalization. The normalization factor *d*_0_ ensures that the TM-score is independent of protein length, and is calculated as:

(Equation 4) $$d_{0}=\left\{ \begin{array}{l} 1.24\sqrt[{3}]{L-15}-1.8,\;if\;L>21\\ 0.5,\;if\;L\leq 21 \end{array}\right.$$

For nucleic acids, *d*_0_ is slightly different:

(Equation 5) $$d_{0}=\left\{ \begin{array}{l} \begin{array}{l} 0.6\sqrt{L-0.5}-2.5,\;if\;L\geq 30\\ 0.7,\;if\;24\leq L\leq 29\\ 0.6,\;if\;20\leq L\leq 23 \end{array}\\ \begin{array}{l} 0.5,\;if\;16\leq L\leq 19\\ 0.4,\;if\;12\leq L\leq 15\\ 0.3,\;if\;L\leq 11 \end{array} \end{array}\right.\;$$

Statistics obtained on inter- and intra-family pairwise alignment show that TM-score≥0.5 (Xu and Zhang, 2010) or ≥0.45 (Gong et al., 2019) corresponds to a pair of proteins or RNAs, respectively, sharing the same topology.

It is NP-hard to identify the superimposition (i.e., rotation and translation of one structure relative to another) that maximize the TM-score. Therefore, the TM-score superimposition given the alignment (i.e., residue level correspondence) is solved numerically by extracting all continuous fragment pairs with length *L*_*ali*_, *L*_*ali*_/2, *L*_*ali*_/4, … , 4. A superimposition is performed for each pair of fragments using the Kabsch algorithm (Kabsch, 1976) to minimize the RMSD. Among these superimpositions, the one corresponding to the highest TM-score is considered the optimal TM-score superimposition.

#### Secondary structure assignment in US-align2

US-align2 has built-in subroutines to assign secondary structures, which is then used to guide initial structure alignments. For proteins, US-align2 assigns one of the four secondary structure states (helix, β-strand, turn, or random coil) to each amino acid based on the inter-atomic distances among five neighboring Cα atoms (Zhang and Skolnick, 2005). Specifically, for residue *i*, we calculate *d*_13_, *d*_14_, *d*_15_, *d*_24_, *d*_25_ and *d*_35_, which correspond to the Cα distances between residues *i*-2 and *i*, *i*-2 and *i*+1, *i*-2 and *i*+2, *i*-1 and *i*+1, *i*-1 and *i*+2, and *i* and *i*+2, respectively. Residue *i* is assigned as part of a helix if $max\left\{\left|d_{15}-6.37\right|,\left|d_{14}-5.18\right|,\left|d_{25}-5.18\right|,\left|d_{13}-5.45\right|,\left|d_{24}-5.45\right|,\left|d_{35}-5.45\right|\right\}<2.1$. It is assigned part of a β-strand if $max\left\{\left|d_{15}-13\right|,\left|d_{14}-10.4\right|,\left|d_{25}-10.4\right|,\left|d_{13}-6.1\right|,\left|d_{24}-6.1\right|,\left|d_{35}-6.1\right|\right\}<1.42$. If the residue belongs to neither a helix nor a β-strand, it is assigned as a turn if $d_{15}<8$. Otherwise, the residue is assigned as part of a random coil.

For RNAs, the secondary structure, i.e., base pairing, is assigned by US-align2 as in our previous work (Gong et al., 2019). For two nucleotides to be considered as forming base pair, they must satisfy the following three conditions. First, the distance between the pair of C3’ atoms should fall within 12.5 to 15.0 Å. Second, only G:C, G:U and A:U pairs are allowed. Third, the singleton pair is excluded, i.e., if neither nucleotide pair *i*-1 and *j*+1, nor nucleotide pair *i*+1 and *j*-1 satisfy the above two criteria, nucleotide *i* and *j* are not considered paired either. Based on these three criteria, a nucleotide can be assigned to one of the three secondary structure state: unpaired, paired with an upstream base, or paired with a downstream base.

#### US-align2 for SQ alignment

Similar to our previous study (Zhang et al., 2022), SQ alignment of US-align2 starts with five different initial alignments, which are based on gapless sliding, secondary structure matching, half-half combination of secondary structure matching and gapless sliding, superimposition of large fragments with length *L*/2 and *L*/3, and superimposition of small fragments with length 4. For each initial alignment, a set of heuristic superimposition-alignment iterations are performed until convergency. In each iteration, a TM-score superimposition is performed based on the alignment obtained from the previous iteration. The new superimposition is used to derive a new alignment using Needleman-Wunsch (NW) global alignment (Needleman and Wunsch, 1970) with a gap penalty of −0.6 and a residue-level TM-score for aligning residue *i* from Structure A to residue *j* from Structure B:

(Equation 6) $$TM_{i,j}=\frac{1}{1+{\left(d_{i}/d_{0}\right)}^{2}}$$

#### US-align2 for fNS alignment

US-align2 fNS alignment starts with an initial alignment that is non-sequential. To construct this alignment, for each residue *i*, we extract the *K* = 5 closest residues in Euclidean distance, including residue *i* itself, to form a fragment with length *K*. The *K*-residue-long fragment for residue *i* from Structure A can then be superimposed by Kabsch algorithm to the fragment for residue *j* from Structure B to calculate the alignment score for the residue pair:

(Equation 7) $$score_{i,j}=\frac{1}{1+{\left(d_{i,j,K}/d_{0}\right)}^{2}}$$

Here, *d*_*i*,*j*,*K*_ is the distance between the *K*-th closest residue to *i* in Structure A and *K*-th closest residue to *j* in Structure B after the superimposition of the two fragments. An NS alignment can then be derived using the Enhanced Greedy Search (EGS) algorithm (Hu et al., 2018) as illustrated by Figure S1. Starting from this initial alignment, a set of heuristic superimposition-alignment iterations are executed until convergency. In each iteration, a TM-score superimposition is performed based on the NS alignment obtained from the previous iteration. The new superimposition is used to derive a new NS alignment using EGS guided by the residue-level TM-score defined by Equation 7.

#### US-align2 for sNS alignment

The sNS alignment process in US-align2 uses the SQ alignment as the initial alignment, followed by the superimposition-alignment iterations until convergency. In each iteration, a TM-score superimposition is performed based on the alignment from the previous iteration. A new alignment is then derived from the superimposition by NW global alignment. This alignment is further refined by the swapping stage of EGS (Figure S1E), where swaps are forbidden if they introduce non-sequential alignments within any secondary structure elements (helices and β-strands for proteins and helices in nucleic acids).

#### Differences among US-align2, TM-align and RNA-align

SQ alignment of US-align2 uses the same iterative superimposition-alignment search algorithm and the same scoring function (TM-score) as TM-align (Zhang and Skolnick, 2005) and RNA-align (Gong et al., 2019) without retuning any hyperparameter, including such as *d*_0_ in (Equation 4), (Equation 5). Therefore, US-align2 SQ results are identical to TM-align and RNA-align for pairwise alignment of monomeric proteins and RNAs, respectively, as the alignment algorithms of all three programs are deterministic. Compared to TM-align and RNA-align, as well as US-align (Zhang et al., 2022) which unifies TM-align and RNA-align into a single program, the major technical advances of US-align2 are the sNS and fNS alignment modes based on the EGS algorithms, which can generate different, usually more sensitive, alignment results despite using the same scoring function.

### Quantification and statistical analysis

For all benchmark studies, error bars indicating standard error of mean (SEM) are indicated as error bars on bar plots.
