# Supplementary material for: A unified approach to sequential and non-sequential structure alignment of proteins, RNAs, and DNAs
Source: iScience. 2022 Sep 28;25(10):105218. doi: 10.1016/j.isci.2022.105218 (PMC9557024; doi:10.1016/j.isci.2022.105218)
Supplement: Document S1. Figure S1 [file mmc1.pdf]

iScience, Volume *25*

Supplemental information

A unified approach to sequential and non-sequential structure alignment of proteins, RNAs, and DNAs

Chengxin Zhang and Anna Marie Pyle

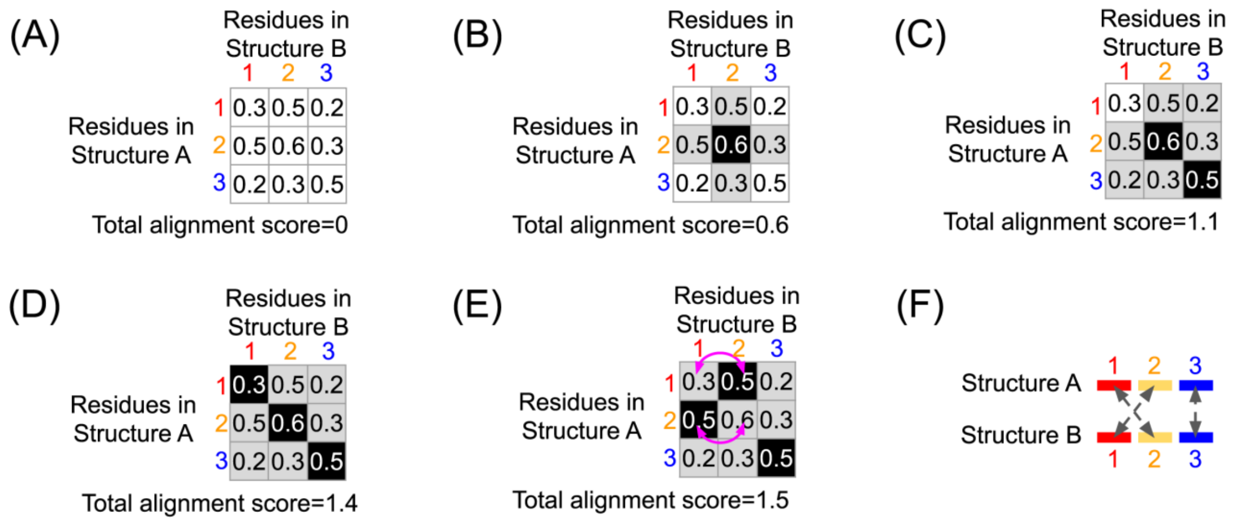

Figure S1. Illustration of NS alignment by Enhanced Greedy Search (EGS) for a pair of structures with *L*=3 residues.

(A) All-against-all residue-to-residue scores were computed and stored as an *L* by *L* matrix. Each row and column corresponded to a residue in Structure A and Structure B, respectively. (B) The residue pair with the highest score (black cell in the middle) was selected as the first assigned pair. Other cells in the same row or the same column (grey cells) were considered invalid assignments. (C) The chain pair with the highest TM-score among the remaining cells was selected as the next assigned chain pair (black cell at lower right). Other cells in the same row or column were marked as invalid (grey). (D) Step C was repeated until no more assignments could be made. (E) In the swapping stage of EGS, for every two assigned chain pairs, the chain assignments were swapped (double arrows) if the swapping led to higher total alignment score. The swapping was repeated until no swap was possible. (F) The final residue pair assignments were indicated by the dashed double arrows.
